# Synchronous double primary malignant tumor of the gallbladder and liver: a case report

**DOI:** 10.1186/1477-7819-9-84

**Published:** 2011-08-03

**Authors:** Ji Won Kim, Jae Woong Han, So Young Jung, Jae Pil Jung, Jeong Won Kim

**Affiliations:** 1 Department of Surgery, Kangnam Sacred Heart Hospital, Hallym Medical Center, 948-1, Daerim-1Dong, Yeongdeunpo-gu, Seoul 150-950, Korea; 2 Department of Pathology, Kangnam Sacred Heart Hospital, Hallym Medical Center, 948-1, Daerim-1Dong, Yeongdeunpo-gu, Seoul 150-950, Korea

**Keywords:** Hepatocellular carcinoma, Gallbladder cancer, synchronous double primary malignant tumor

## Abstract

We report a case of synchronous double primary tumor of gallbladder and liver. A 63-year-old male was admitted to the hospital complaining of abdominal discomfort. Enhanced computed tomography of the abdomen showed acute cholecystitis with tiny gallbladder stones and a 2.2 cm size enhanced nodule in the left lobe of the liver. Under the impression of acute cholecystitis with gall bladder stones and hepatocellular carcinoma of the left Liver, the patient underwent a laparotomy. At laparotomy, a mass was palpated on the surface of the neck portion of the gall bladder. Intraoperative frozen diagnosis revealed adenocarcinoma of the gall bladder. The patient was diagnosed as having gall bladder cancer and hepatocellular carcinoma, so extended cholecystectomy with dissection of regional lymph nodes and left hemihepatectomy were performed. Histological examination revealed moderated differentiated adenocarcinoma of gallbladder and hepatocellular carcinoma of liver. To our knowledge, the simultaneous occurrence of primary malignant tumor of the gallbladder and liver has never been published before. The patient is doing well with no evidence of recurrence 17 months after surgery.

## Background

Synchronous double primary malignant neoplasms are a secondary malignancy occurring at the same time or within 6 months after the first malignancy. Improvement of survival rates for patients with malignancy due to early diagnosis and new treatments has enabled more patients to survive long enough to develop the subsequent primary malignancy, and development of more sophisticated diagnostic tools has made possible the detection of synchronous occult malignancies. Synchronous double primary malignant neoplasms in a single patient have been well-documented in the literature. But, synchronous double primary malignant tumor of gallbladder and liver has never been reported. Herein, the authors report a case of a 63-year-old male patient with double primary cancer of gallbladder and liver.

## Case presentation

**Figure 1:**
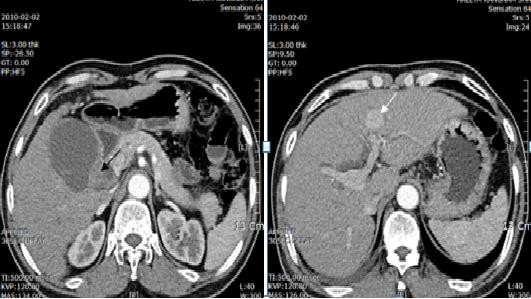
**CT imaging results**. a) A distended gallbladder with wall thickening and tiny gallstones in the gallbladder (black arrow) is evident. b) A tumorous lesion heterogeneously enhanced by contrast media in the left lobe of the liver (white arrow) is evident.

**Figure 2:**
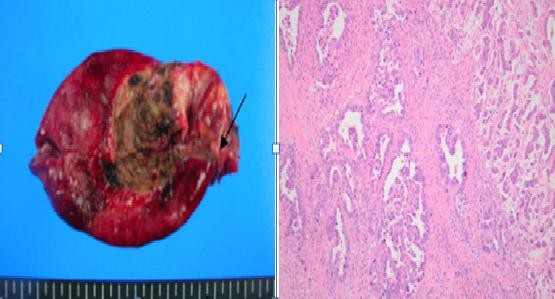
**Gross and histological appearance of the gall bladder**. a). Gross appearance of gall bladder. Mass was palpated on the surface of neck portion of gall bladder (black arrow). b). Moderately differentiated adenocarcinoma with prominent desmoplastic response (right side) infiltrating the gallbladder wall. Invasive micropapillary components (left side) are evident. (Hematoxylin and eosin stain, × 100)

**Figure 3:**
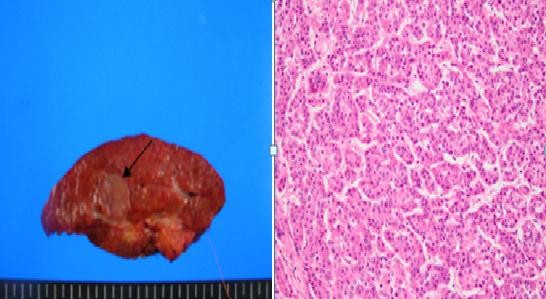
**Gross and histological appearance of the liver**. a). The cut section, showing a brownish, ovoid, and highly circumscribed mass measuring 2.2 × 2.0 cm (black arrow). The remaining parenchyma is diffusely cirrhotic, and in which the cirrhotic nodules measure from 0.2 cm to 1 cm in diameter. b). Hepatocellular carcinoma with trabecular pattern is noted. Tumor cell cords are separated by sinusoid-like blood spaces. (Hematoxylin and eosin stain, × 200)

In February 2010, a 63-year-old male patient visited our hospital with the chief complaint of abdominal discomfort in right upper quadrant for 1 year. In 2008, the patient had been diagnosed with acute cholecystitis at our hospital. There was no remarkable family history. On admission, vital signs (blood pressure, heart rate, respiration rate, and body temperature) were within normal limits. The patient was in good general health and had no significant weight loss. On physical examination, the conjunctiva was anemic. The abdomen was soft but tender in the right upper quadrant. Slight resistance, but no rigidity, was recognized in the tender area. Complete blood count and serum biochemistry data on admission showed the following: white blood cell, 16,120/uL; hemoglobin, 8.5 g/dl; hematocrit, 25.3%; platelet, 178000/mm^3^; blood glucose, 209 mg/dl; total bilirubin, 0.5 mg/dl; alkaline phosphatase (ALP), 41 IU/l; aspartate aminotransferase (AST), 193 IU/l; alanine aminotransferase (ALT), 146 IU/l, and amylase 212 IU/l; C-reactive protein 76.7 mg/l. Viral markers were hepatitis B surface antigen [HBsAg(+)], anti-HBs(-) and anti-hepatitis C virus(-). Tumor marker assays showed alpha-fetoprotein was 17.9 n/ml (normal 0-8.1), carcinoembryonic antigen (CEA) was 4.2 ng/ml (normal 0-5), carbohydrate antigen 19-9 (CA 19-9) was 112.5 U/ml (normal 0-37). A computed tomography (CT) scan of the abdomen showed distension of the gallbladder with gallbladder stones and gallbladder wall thickening, suggesting acute cholecystitis (Figure 1a), cirrhosis of liver and heterogenously enhanced tumorous lesion in the left lobe of liver (Figure 1b). Thus, the preoperative diagnosis was hepatocellular carcinoma and acute cholecystitis accompanied by gallstones. At laparotomy, the gallbladder was slightly distended and showed wall thickening. There was a palpable mass felt on the surface of the gallbladder neck portion. The patient underwent surgical resection of the gallbladder and the left lobe of the liver. Intraoperative histologic examination revealed adenocarcinoma of gallbladder with invasion to the perimuscular connective tissue. The patient was diagnosed with synchronous double primary cancer of the gallbladder and liver, so additional resection of extrahepatic bile duct and segment 5 of liver, with a dissection of regional lymph nodes, was performed. Biliary reconstruction was performed by Roux-en-Y hepaticojejunostomy. The resected gallbladder was 12 cm in length and 13 cm in greatest circumference, and contained multiple black pigment stones (Figure 2a). The mass in the neck portion of gallbladder was 5 × 3 × 1.5 cm in size and microscopic findings revealed moderate adenocarcinoma with prominent desmoplastic response infiltrating the gallbladder wall. There were invasive micropapillary components (Figure 2b). The tumor in the left lobe of liver was about 2.2 × 2.0 × 1.5 cm in size, the cut section of which revealed a brownish, ovoid, and highly-circumscribed mass (Figure 3a). Microscopically, the tumor in the lateral segment of liver was a trabecular hepatocellular carcinoma with grade II-III cellular atypism in the Edmondson classification. Neither portal or hepatic vein infiltration, nor biliary duct infiltration was found (Figure 3b). There were no microscopically evident malignant cells in surgical resection margins (extended cholecystectomy, Lt. hemihepatectomy). The postoperative course was uneventful except for postoperative ascites, which was successfully treated conservatively. The patient was discharged on postoperative day 31. We planned adjuvant chemoradiation therapy because the gallbladder cancer presented as a T2 lesion with perineural invasion, but it was not performed due to the patient's refusal. The patient has been under regular follow-up with clinical examination and liver function test every two months, with surveillance for tumor markers (AFP, CA19-9, CEA) and abdominal CT scan being done at 4 months intervals (last follow-up June 2011). The patient is doing well with no evidence of local or distant recurrence more than 17 months after surgery.

## Discussion

Multiple primary malignant tumors in a single patient are relatively rare. In reviews of the literature regarding multiple primary malignant tumors, the overall occurrence rate of multiple primary malignancies is between 0.73% and 11.7% [1]. Multiple primary cancers have become more common because of an increase in the number of elderly patients and advancement in diagnostic techniques. Three diagnostic criteria have been proposed for multiple primary malignancy: 1) each tumor must present definite features of malignancy, 2) each must be distinct, and 3) the chance of one being a metastasis of the other must be excluded [2]. Multiple primary cancers may be synchronous or metachronous depending on the interval between their diagnosis. Synchronous cancers are second tumors occurring simultaneously or within 6 months after the first malignancy, while metachronous multiple malignancies are secondary cancers that developed after more than 6 months after from the first malignancy [3]. Multiple primary malignancies are classified into four types: 1) multicentric, if the two distinct carcinoma arise in the same organ or tissue; 2) systemic, if they arise on anatomically or functionally allied organs of the same system (colon and rectum cancers), 3) paired organs, as in the breasts, and 4) random, if they occur as a co-incidental or accidental association in unrelated sites [4]. In our patient, the malignant features were histopathologically proven in each tumor. Each tumor was pathologically categorized as a different type; namely, the one detected in the gall bladder was a moderately differentiated adenocarcinoma, the one in the liver was a hepatocellular carcinoma. These findings might also support the fact that these two cancers occurred in a random and synchronous manner. There was one case reported in the literature who had synchronous triple primary cancers of gallbladder, common bile duct and liver in a women with primary biliary cirrhosis [5]. In our case, two different type malignant tumors, adenocarcinoma of gallbladder and hepatocellular carcinoma, were observed in a male patient without primary biliary cirrhosis. Hepatocelullar carcinoma is known to be pathogenically associated with liver cirrhosis, chronic hepatitis virus infection, and abuse of alcohol [6]. Gallbladders containing stones or infectious agents develop cancer as a result of recurrent trauma and chronic inflammation. Although the mechanism involved in the development of multiple primary cancer has not been clarified, some factors such as heredity, constitution, environmental and immunological factors, carcinogenic, viruses, radiological and chemical treatments have been implicated [7]. In the present case, gallbladder stones and chronic hepatitis B virus infection may have played an important role in the pathogenesis of gallbladder adenocarcinoma and hepatocellular carcinoma, respectively. The prognosis of patients with multiple primary malignant tumors can be determined independently by the stage of each malignancies. In the present case, adenocarcinoma of gall bladder was T2N0M0 (stage II) and hepatocellular carcinoma was T1N0M0 (stage I). The surgical treatment of choice for synchronous multiple primary malignancies is curative resection of each malignant tumors [8].

## Conclusions

We report the first case of synchronous double primary malignancies of gallbladder and liver. The possibility of synchronous multiple primary malignancies should be noted in the treatment of elderly patients with malignant tumor. Multiplicity of primary malignancies itself does not necessarily indicate a poor prognosis as long as adequate diagnosis and treatment are performed.

## Consent

Written informed consent was obtained from the patient for publication of this case report and any accompanying images. A copy of the written consent is available for review by the Editor-in-Chief of this journal.

## Competing interests

The authors declare that they have no competing interests.

## Authors' contributions

Ji Won Kim, Jae Pil Jung, Jae Woong Han, So Young Jung and Jeong Won Kim made up the surgical and pathological team involved in the case. Jae Pil Jung and Ji Won Kim wrote and edited the manuscript. All authors read and approved the final manuscript.
